# There was a similar U-shaped nonlinear association between waist-to-height ratio and the risk of new-onset hypertension: findings from the CHNS

**DOI:** 10.3389/fnut.2023.1304521

**Published:** 2023-12-14

**Authors:** Zhenwei Wang, Qian Shi, Xuejiao Yan, Junnan Tang, Jinying Zhang

**Affiliations:** ^1^Department of Cardiology, The First Affiliated Hospital of Zhengzhou University, Zhengzhou, China; ^2^Key Laboratory of Cardiac Injury and Repair of Henan Province, Zhengzhou, China; ^3^Henan Province Clinical Research Center for Cardiovascular Diseases, Zhengzhou, China; ^4^Neonatal Intensive Care Unit, The Third Affiliated Hospital of Zhengzhou University, Zhengzhou, China; ^5^Department of Cardiology, The Affiliated Changzhou No.2 People's Hospital of Nanjing Medical University, Changzhou, China

**Keywords:** waist-to-height ratio, body mass index, hypertension, incidence, prevalence

## Abstract

**Background:**

The association between waist-to-height ratio (WHtR) with hypertension has not been adequately explained, so in this study we sought to clarify the predictive role of WHtR on the incidence of hypertension as well as the potential nonlinear associations in the general population.

**Methods:**

In this large prospective cohort study, a total of 4,458 individuals from the China Health and Nutrition Survey (CHNS) were included in the analysis. Multivariate Cox regression analyses, subgroup analyses, receiver operator characteristic (ROC) and restricted cubic spline (RCS) analyses were used to examine the association of WHtR with the risk of new-onset hypertension.

**Results:**

Hypertension occurred in 32.8% of participants during the maximum six-year follow-up period. Compared with the group with lower WHtR, the group with higher WHtR had a higher incidence of hypertension (*p* < 0.001). Multivariate Cox regression analysis showed that the risk of hypertension was 1.45 times higher in the high WHtR group than in the low WHtR group, and that the risk of hypertension increased by 30.4% for every 0.1 unit increase in WHtR (*p* < 0.001). Subgroup analyses also validated the stratified associations between WHtR and the risk of new-onset hypertension in most subgroups (*p* < 0.05). ROC analyses also revealed that WHtR was superior to body mass index in predicting new-onset hypertension (AUC: 0.626 vs. 0.607, *p* = 0.009). Further RCS analysis detected a nonlinear association between WHtR and risk of new-onset hypertension (P for nonlinearity <0.001).

**Conclusion:**

WHtR was nonlinearly associated with the risk of new-onset hypertension in the general population.

## 1 Introduction

With the development of society and the improvement of economic conditions, hypertension has gradually become one of the most important public health problems worldwide (1). Current evidence suggests that more than 1.5 billion people will bear the health and economic burden of hypertension in the coming years, which predicts that a range of diseases attributable to hypertension will also be a constant threat to public health in everyday life, particularly cardiovascular disease (CVD) and chronic kidney disease (CKD) (2). Multiple studies from 2016 to 2023 have shown that the global disease burden of CVD, CKD, and cardiovascular death due to hypertension continues to increase, with a study from southwestern China predicting that a 25% reduction in the prevalence of high systolic blood pressure (SBP) by 2030 would result in a reduction of 26,200 and 8,000 cases of CVD and CKD, respectively, and a reduction of cardiovascular mortality and CKD mortality by 32.8 and 16%, respectively (3–8). These data suggest that the burden of a series of diseases caused by hypertension remains very heavy, and the formulation of high-quality prevention, management and treatment strategies for hypertension often fail to address the underlying problem, so early screening and intervention of controllable risk factors for hypertension from the source is particularly important for reducing the incidence, prevalence and mortality of hypertension and related diseases.

Currently widely recognized risk factors for hypertension are metabolically related risk factors such as uneven fat distribution and overnutrition, in addition to age, physical inactivity, stress, genetic factors, unhealthy diet and environment (such as noise, environmental pollution and occupational exposure) (9–11). There are several anthropometric indicators that can effectively reflect the nutritional status of the body, among which waist-to-height ratio (WHtR) seems to be superior to waist circumference (WC), waist-to-hip ratio (WHR) and body mass index (BMI) in predicting some metabolism-related diseases, such as diabetes, diabetic peripheral neuropathy, and CVD (12–15). In addition, several studies have confirmed the correlation between WHtR and the prevalence and incidence of hypertension (16–19). For example, Rezende et al. found that WHtR could be used as an independent risk factor for new-onset hypertension in a small sample cohort study (16). And Moosaie et al. also found that WHtR was a more accurate tool to predict the incidence of hypertension in patients with type 2 diabetes in another large cohort study (17). However, the existence of stable stratified and nonlinear associations between WHtR and the risk of new-onset hypertension and whether WHtR improves the prediction of hypertension risk still need to be fully explained. Therefore, based on the current research background, we explored the correlation between WHtR and new-onset hypertension as well as potential stratified associations and nonlinearities in this large prospective cohort study based on a Chinese community-based population.

## 2 Subjects, materials and methods

### 2.1 Study population

In this large longitudinal cohort study with a convenience sample, all participants were from the 2009 China Health and Nutrition Survey (CHNS 2009). Inclusion criteria were shown as follows: (1) age ≥ 18 years; and (2) complete baseline data. The exclusion criteria were as follows: (1) participants with hypertension at baseline; (2) lack of WHtR data at baseline; and (3) lack of follow-up data. All participants signed an informed consent form at the time of enrolment in the CHNS. The study protocol regarding the CHNS was in accordance with the Declaration of Helsinki and was approved by the institutional review committees at the University of North Carolina at Chapel Hill and the National Institute of Nutrition and Food Safety, Chinese Center for Disease Control and Prevention.

### 2.2 Data collection and definitions

In this study, we downloaded the required publicly available anonymised data from the CHNS website and merged and cleaned them for statistical testing, including demographic data, anthropometric assessments, comorbidities, medication data, blood markers and follow-up data. The area of residence was classified into two categories: suburban or rural village and urban. Education level was categorised as lower than high school completed, high school completed and higher than high school completed. Marital status was categorised as married and unmarried. Smoking status was categorised as present smoker, past smoker and never smoker, and frequency of alcohol consumption was categorised as every day, 3–4 times per week, 1–2 times per week and ≤ 2 times per month (20, 21). Diabetes was defined as fasting plasma glucose (FPG) ≥ 7.0 mmol/L, hemoglobin A1c ≥ 6.5%, use of glucose-lowering medication, or previous diagnosis of diabetes (22).

Anthropometry was measured by a physician, nurse, health worker or other health professional according to standard procedures, where height (in the unshod state) was measured using a portable altimeter with an accuracy of 0.1 cm, weight (in the lightly clothed state) was measured using a calibrated beam scale with an accuracy of 0.1 kg, and WC (in the naturally upright respiratory state) was measured using a non-elastic tape measure in the midpoint level between the lowest rib and the iliac crest, with an accuracy of 0.1 cm. In this study, BMI was defined as weight (kg) divided by height (m^2^). WHtR was defined as WC (cm) divided by height (cm) and categorized into three groups based on tertiles: ≤ 0.47 (T1: low WHtR), 0.47–0.53 (T2: medium WHtR), and > 0.53 (T3: high WHtR) (23). Besides, our study population was a community-based population from China, so we defined obesity as BMI ≥ 28 kg/m^2^ according to Chinese criteria and not according to the WHO criteria (BMI ≥ 30 kg/m^2^) (24, 25).

All participants were followed up from 2009 to 2015. New-onset hypertension was assessed based on a combination of blood pressure, medical history, and medication information, that is, SBP ≥ 140 mmHg and/or diastolic blood pressure (DBP) ≥ 90 mmHg or diagnosed by a physician or under antihypertensive treatment during the follow-up (26). All participants were asked to sit and rest for at least 10 min before taking blood pressure measurements, then three blood pressure measurements were taken by trained staff according to the standard protocol and using a suitable cuff and mercury sphygmomanometer, with at least 1 min between measurements (27, 28). In this study, the values of SBP and DBP were obtained by averaging the three blood pressure measurements.

In addition, blood specimens were required to be fasted for 8–12 h before they could be collected. All laboratories fulfill all requirements for measurement and testing accuracy. Blood marker data included triglycerides (TG), total cholesterol (TC), low-density lipoprotein cholesterol (LDL-C), high-density lipoprotein cholesterol (HDL-C), apolipoprotein A1 (ApoA1), apolipoprotein B (ApoB), lipoprotein(a), creatinine, uric acid, FPG, HbA1c and high-sensitivity C-reactive protein (Hs-CRP). The 12 mL of blood was drawn from the upper extremity vein of each participant by trained professionals and placed into three tubes and sent to three standardized laboratories for measurement by specialized technicians respectively, in which FPG was measured by glucose oxidase in the local laboratory, TG and TC were measured by enzymatic process (dissociation) and enzymatic process (direct), respectively, in the local laboratory, HDL-C and LDL-C were measured by the enzymatic process (direct) in the Beijing central laboratory (the China-Japan Friendship Hospital, Ministry of Health laboratory), HbA1c was measured by the high-performance liquid chromatography in the provincial laboratory, ApoA1, ApoB and lipoprotein(a) were measured by the immunoturbidimetry in the Beijing central laboratory, and creatinine, uric acid and Hs-CRP were determined by picric acid, uric acid-POD and latex agglutination, respectively, in the Beijing central laboratory. The specifics of the above questionnaires and marker measurements can be reviewed elsewhere (20, 21).

### 2.3 Statistical analysis

In this study, we presented continuous variables that conformed to the normal distribution as mean ± standard deviation and used independent sample t-tests and one-way ANOVA test to examine the differences between groups, whereas continuous variables with the skewed distribution were presented as median (first quarter, third quarter) and non-parametric tests were used to assess the differences between groups, and then the categorical data were presented as frequency (percentage) and the differences between groups were evaluated by Chi-square test or Fisher’s exact test. Univariate Cox regression models were used to test the correlation of each variable with the risk of hypertension and three multivariate Cox regression models were constructed stepwise to test the independent correlation of WHtR with the risk of hypertension. Model 1 included age and sex; model 2 included age, sex, suburban or rural village, educational level, married, smoking status, drinking status, diabetes and hypoglycemic drugs; model 3 included all variables in model 2 as well as BMI, SBP, DBP, TC, TG, LDL-C, ApoB, creatinine, uric acid, FPG, HbA1c and Hs-CRP. Participants were then classified into 16 subgroups based on the 8 variables and further examined for stratified association between the WHtR and risk of hypertension as well as the interaction between WHtR and stratified variables by likelihood ratio tests in multivariate Cox regression models. Receiver operator characteristic (ROC) analyses were used to test the predictive ability of height, weight, WC, BMI, and WHtR for hypertension and to assess whether the diagnostic performance of WHtR was superior to that of other indicators. Finally, restricted cubic spline (RCS) analysis was used to explore the nonlinear association between WHtR and hypertension risk. All statistical tests were performed using the SPSS 26.0, MedCalc 19.6.1, GraphPad Prism 8 or R 3.6.3 and a two-tailed *p* value <0.05 was considered statistically significant.

## 3 Results

### 3.1 Baseline characteristics

**Figure 1:**
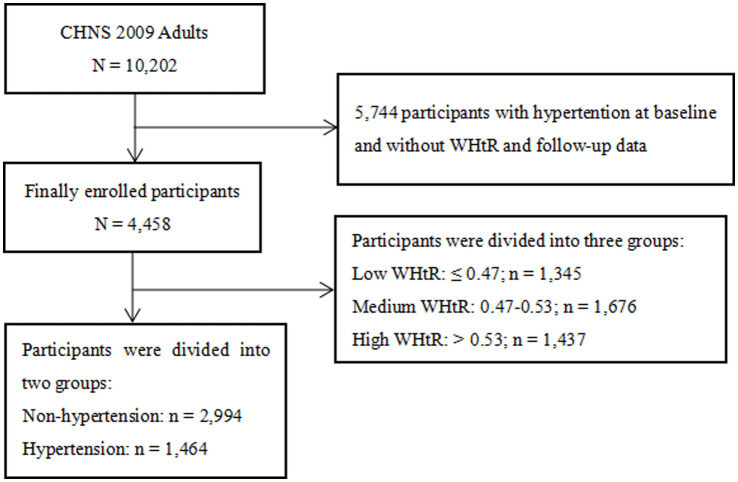
Flow chart of the study population. CHNS, China Health and Nutrition Survey; WHtR, waist-to-height ratio.

**Figure 2:**
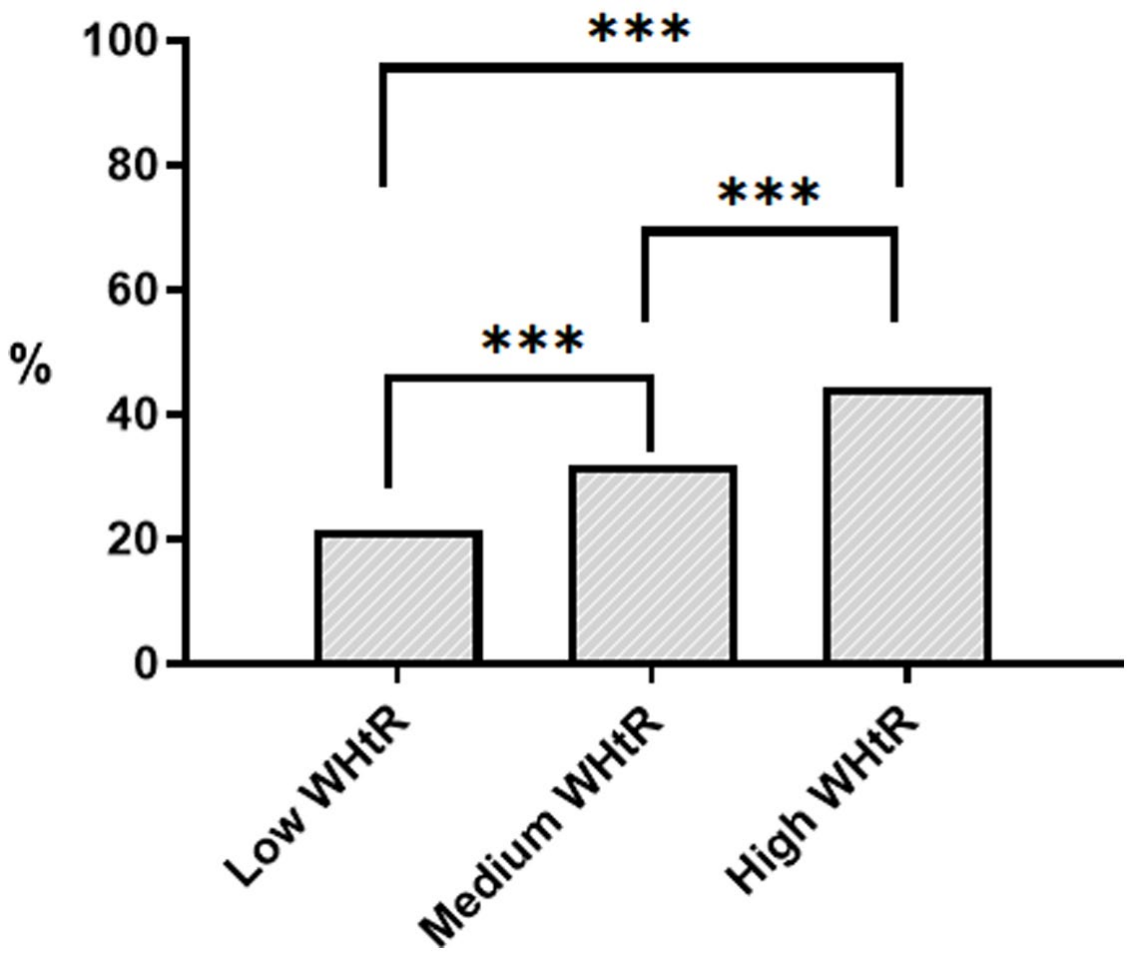
Histogram of the incidence of hypertension grouped by WHtR. *** *p* < 0.001. WHtR, waist-to-height ratio. Low WHtR ≤0.47; Medium WHtR: 0.47–0.53; High WHtR >0.53.

After excluding individuals without baseline WHtR and follow-up data, we included 4,458 participants from the CHNS 2009, of whom 1,464 (32.8%) had new-onset hypertension (Figure 1). In Table 1, compared with participants without new-onset hypertension, participants with new-onset hypertension were more likely to be older, male, suburban or rural village dwellers, less educated, smokers, drinkers, have diabetes and hypoglycemic drugs users. And levels of weight, WC, BMI, WHtR, SBP, DBP, TG, TC, LDL-C, ApoB, uric acid, FPG, HbA1c and Hs-CRP were also higher in participants with new-onset hypertension (*p* < 0.05). Table 2 and Figure 2 showed that the incidence of hypertension was significantly higher in participants with higher WHtR than in those with lower WHtR (*p* < 0.001). And compared to individuals with lower WHtR, individuals with higher WHtR were more likely to be older, females, less educated, never-smokers, have diabetes, and use hypoglycemic drugs (*p* < 0.05). In addition, individuals with higher WHtR also had higher levels of weight, WC, BMI, SBP, DBP, TG, TC, LDL-C, ApoB, uric acid, FPG, HbA1c, and Hs-CRP, and lower levels of height, HDL-C, ApoA1, and creatinine (p < 0.05).

**Table 1:** Baseline characteristics of participants stratified by the new-onset hypertension.

	Total population	Non-hypertension	Hypertension	P value
Age, years	47.82 ± 13.35	45.33 ± 13.02	52.92 ± 12.53	< 0.001
Sex, male, *n* (%)	2071 (46.50%)	1,332 (44.50%)	739 (50.50%)	< 0.001
Suburban or rural village, *n* (%)	3,270 (73.40%)	2,163 (72.20%)	1,107 (75.60%)	0.017
Educational level, *n* (%)				< 0.001
Lower than high school	3,410 (76.60%)	2,232 (74.70%)	1,178 (80.50%)	
High School	546 (12.30%)	386 (12.90%)	160 (10.90%)	
Higher than high school	497 (11.20%)	371 (12.40%)	126 (8.60%)	
Married, *n* (%)	3,945 (88.70%)	2,647 (88.60%)	1,298 (88.90%)	0.777
Smoking status, *n* (%)				0.018
Present	1,313 (29.50%)	852 (28.50%)	461 (31.50%)	
Past	106 (2.40%)	63 (2.10%)	43 (2.90%)	
Never	3,037 (68.20%)	2077 (69.40%)	960 (65.60%)	
Drinking status, *n* (%)				< 0.001
Every day	397 (26.60%)	215 (22.50%)	182 (34.00%)	
3–4 times/week	214 (14.30%)	132 (13.80%)	82 (15.30%)	
1–2 times/week	362 (24.30%)	228 (23.80%)	134 (25.00%)	
≤ 2 times/month	519 (34.80%)	382 (39.90%)	137 (25.60%)	
Diabetes, *n* (%)	307 (6.90%)	168 (5.60%)	139 (9.50%)	< 0.001
Hypoglycemic drugs, *n* (%)	53 (1.20%)	25 (0.80%)	28 (1.90%)	0.002
Height, cm	161.18 ± 8.39	161.16 ± 8.26	161.23 ± 8.67	0.797
Weight, cm	59.80 ± 10.65	58.74 ± 10.07	61.98 ± 11.46	< 0.001
WC, cm	81.25 ± 9.72	79.85 ± 9.30	84.13 ± 9.92	< 0.001
WHtR	0.51 ± 0.06	0.50 ± 0.06	0.52 ± 0.06	< 0.001
BMI, kg/m^2^	22.94 ± 3.21	22.55 ± 3.06	23.75 ± 3.35	< 0.001
SBP, mmHg	116.58 ± 11.21	114.53 ± 11.03	120.99 ± 10.28	< 0.001
DBP, mmHg	76.08 ± 7.46	74.98 ± 7.54	78.43 ± 6.73	< 0.001
TG, mmol/L	1.19 (0.81, 1.81)	1.13 (0.78, 1.72)	1.33 (0.91, 2.02)	< 0.001
TC, mmol/L	4.80 ± 0.96	4.73 ± 0.93	4.95 ± 1.00	< 0.001
LDL-C, mmol/L	2.93 ± 0.91	2.87 ± 0.86	3.05 ± 1.01	< 0.001
HDL-C, mmol/L	1.44 ± 0.44	1.45 ± 0.44	1.44 ± 0.44	0.442
ApoA1, g/L	1.16 ± 0.37	1.15 ± 0.35	1.17 ± 0.41	0.079
ApoB, g/L	0.89 ± 0.25	0.87 ± 0.24	0.93 ± 0.26	< 0.001
Lp(a), mg/L	79.00 (40.00, 168.00)	77.00 (39.00, 168.00)	83.00 (42.00, 168.00)	0.187
CR, umol/L	86.17 ± 21.41	85.78 ± 23.64	86.94 ± 16.05	0.106
Uric acid, umol/L	299.13 ± 101.14	294.71 ± 103.79	307.95 ± 95.08	< 0.001
FPG, mmol/L	5.27 ± 1.27	5.18 ± 1.15	5.45 ± 1.46	< 0.001
HbA1c, %	5.53 ± 0.78	5.47 ± 0.69	5.67 ± 0.91	< 0.001
Hs-CRP, mg/L	1.00 (0, 2.00)	1.00 (0, 2.00)	1.00 (1.00, 2.00)	< 0.001

Data were expressed as mean ± SD, median (interquartile range), or n (%). WC, waist circumference; WHtR, waist-to-height ratio; BMI, body mass index; SBP, systolic blood pressure; DBP, diastolic blood pressure; TG, triglycerides; TC, total cholesterol; LDL-C, low-density lipoprotein cholesterol; HDL-C, high-density lipoprotein cholesterol; ApoA1, apolipoprotein A1; ApoB, apolipoprotein B; Lp(a), lipoprotein(a); CR, creatinine; FPG, fasting plasma glucose; HbA1c, hemoglobin A1c; Hs-CRP, high-sensitivity C-reactive protein.

**Table 2:** Baseline characteristics of participants stratified by the tertiles of WHtR.

	T1	T2	T3	*p* value
Age, years	43.42 ± 14.13	48.57 ± 12.45	51.07 ± 12.48	< 0.001
Sex, male, *n* (%)	692 (51.40%)	829 (49.50%)	550 (38.30%)	< 0.001
Suburban or rural village, *n* (%)	997 (74.10%)	1,228 (73.30%)	1,045 (72.70%)	0.701
Educational level, *n* (%)				< 0.001
Lower than high school	964 (71.80%)	1,263 (75.40%)	1,183 (82.40%)	
High School	188 (14.00%)	212 (12.70%)	146 (10.20%)	
Higher than high school	190 (14.20%)	200 (11.90%)	107 (7.50%)	
Married, *n* (%)	1,138 (85.00%)	1,523 (91.00%)	1,284 (89.50%)	< 0.001
Smoking status, *n* (%)				< 0.001
Present	440 (32.70%)	523 (31.20%)	350 (24.40%)	
Past	26 (1.90%)	44 (2.60%)	36 (2.50%)	
Never	879 (65.40%)	1,108 (66.10%)	1,050 (73.10%)	
Drinking status, *n* (%)				0.243
Every day	111 (24.20%)	173 (28.80%)	113 (26.20%)	
3–4 times/week	65 (14.20%)	79 (13.10%)	70 (16.20%)	
1–2 times/week	104 (22.70%)	149 (24.80%)	109 (25.20%)	
≤ 2 times/month	179 (39.00%)	200 (33.30%)	140 (32.40%)	
Diabetes, *n* (%)	43 (3.20%)	92 (5.50%)	172 (12.00%)	< 0.001
Hypoglycemic drugs, *n* (%)	8 (0.60%)	13 (0.80%)	32 (2.20%)	< 0.001
Height, cm	162.60 ± 8.10	161.60 ± 8.25	159.38 ± 8.52	< 0.001
Weight, cm	53.91 ± 8.31	59.85 ± 9.31	65.27 ± 11.14	< 0.001
WC, cm	71.11 ± 5.04	80.82 ± 4.80	91.26 ± 6.77	< 0.001
BMI, kg/m^2^	20.31 ± 2.08	22.80 ± 2.16	25.58 ± 2.99	< 0.001
SBP, mmHg	113.25 ± 11.38	116.83 ± 10.89	119.31 ± 10.63	< 0.001
DBP, mmHg	74.10 ± 7.76	76.15 ± 7.44	77.78 ± 6.75	< 0.001
TG, mmol/L	0.97 (0.70, 1.32)	1.22 (0.83, 1.79)	1.46 (0.99, 2.39)	< 0.001
TC, mmol/L	4.51 ± 0.90	4.85 ± 0.95	5.00 ± 0.97	< 0.001
LDL-C, mmol/L	2.69 ± 0.82	3.00 ± 0.95	3.07 ± 0.91	< 0.001
HDL-C, mmol/L	1.53 ± 0.47	1.46 ± 0.43	1.35 ± 0.39	< 0.001
ApoA1, g/L	1.19 ± 0.49	1.17 ± 0.31	1.11 ± 0.30	< 0.001
ApoB, g/L	0.79 ± 0.23	0.90 ± 0.25	0.96 ± 0.25	< 0.001
Lp(a), mg/L	79.00 (40.00, 164.50)	84.00 (43.00, 175.00)	73.00 (37.00, 160.00)	0.005
CR, umol/L	87.15 ± 16.30	86.96 ± 28.63	84.39 ± 14.73	0.001
Uric acid, umol/L	286.50 ± 95.03	296.58 ± 92.07	313.42 ± 113.86	< 0.001
FPG, mmol/L	5.01 ± 0.95	5.20 ± 1.16	5.57 ± 1.55	< 0.001
HbA1c, %	5.35 ± 0.66	5.51 ± 0.73	5.73 ± 0.87	< 0.001
Hs-CRP, mg/L	1.00 (0, 1.00)	1.00 (0, 2.00)	1.00 (1, 3.00)	< 0.001
New-onset hypertension, %	289 (21.50%)	536 (32.00%)	639 (44.50%)	< 0.001

Data were expressed as mean ± SD, median (interquartile range), or n (%). WHtR, waist-to-height ratio; WC, waist circumference; BMI, body mass index; SBP, systolic blood pressure; DBP, diastolic blood pressure; TG, triglycerides; TC, total cholesterol; LDL-C, low-density lipoprotein cholesterol; HDL-C, high-density lipoprotein cholesterol; ApoA1, apolipoprotein A1; ApoB, apolipoprotein B; Lp(a), lipoprotein(a); CR, creatinine; FPG, fasting plasma glucose; HbA1c, hemoglobin A1c; Hs-CRP, high-sensitivity C-reactive protein.

### 3.2 Multivariate-adjusted correlation of WHtR with incidence of hypertension

Table 3 presented the multivariate adjusted correlation between WHtR and risk of hypertension. After adjusting for age, sex, residence, educational level, marital status, smoking status, drinking status, diabetes, hypoglycemic drugs, BMI, SBP, DBP, TC, TG, LDL-C, ApoB, creatinine, uric acid, FPG, HbA1c and Hs-CRP, higher WHtR remained strongly associated with higher risk of hypertension [T3 vs. T1, hazard ratio (HR): 1.45, 95% confidence interval (CI): 1.26–1.67, *p* < 0.001; per 0.1-unit increase, HR: 1.30, 95% CI: 1.19–1.43, p < 0.001].

**Table 3:** Multivariate COX regression analysis of association between WHtR and incidence of hypertension.

	Model 1		Model 2		Model 3	
	HR (95% CI)	*p* value	HR (95% CI)	*p* value	HR (95% CI)	*p* value
T1	Ref	–	Ref	-	Ref	–
T2	1.04 (0.90, 1.21)	0.564	1.05 (0.91, 1.21)	0.541	1.02 (0.88, 1.18)	0.801
T3	1.58 (1.37, 1.82)	< 0.001	1.58 (1.37, 1.82)	< 0.001	1.45 (1.26, 1.67)	< 0.001
P for trend	-	< 0.001	–	< 0.001	–	< 0.001
WHtR^a^	1.41 (1.29, 1.53)	< 0.001	1.41 (1.29, 1.53)	< 0.001	1.30 (1.19, 1.43)	< 0.001

a The HR was examined by per 0.1-unit increase of WHtR. Model 1: adjusted for age and sex; Model 2: adjusted for age, sex, suburban or rural village, educational level, married, smoking status, drinking status, diabetes and hypoglycemic drugs; Model 3: adjusted for variables included in Model 2 and BMI, SBP, DBP, TC, TG, LDL-C, ApoB, CR, uric acid, FPG, HbA1c and Hs-CRP. WHtR, waist-to-height ratio; BMI, body mass index; SBP, systolic blood pressure; DBP, diastolic blood pressure; TC, total cholesterol; TG, triglycerides; LDL-C, low-density lipoprotein cholesterol; ApoB, apolipoprotein B; CR, creatinine; FPG, fasting plasma glucose; HbA1c, hemoglobin A1c; Hs-CRP, high-sensitivity C-reactive protein; HR, hazard ratio; CI, confidence interval.

**Figure 3:**
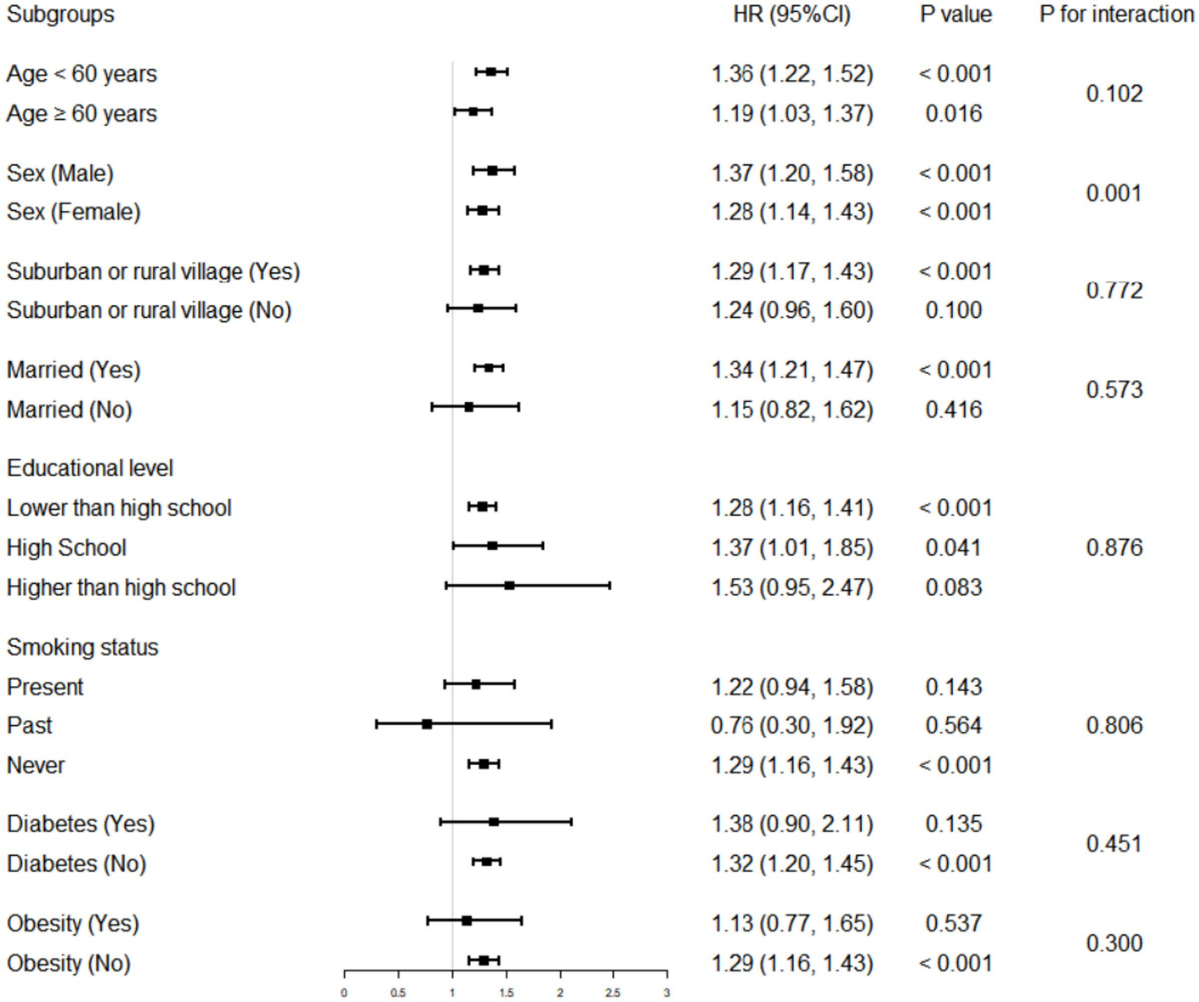
Association of WHtR with the incidence of hypertension in various stratifications. The HR was examined by per 0.1-unit increase of WHtR. The interaction of WHtR and variables used for stratification was examined by likelihood ratio tests. WHtR, waist-to-height ratio; HR, hazard ratio; CI, confidence interval.

Table 4 and Figure 3 presented the results of the subgroup analyses, in which the multivariate-adjusted model adjusted for all the variables contained in model 3 in Table 3, but did not include hierarchical variables as categorical variables. In the subgroups of age < 60 years or ≥ 60 years, male or female, suburban or rural village, married, lower than high school, high school, never smoking, without diabetes, and without obesity, the association between higher levels of WHtR and higher risk of hypertension remained robust (*p* < 0.05). In addition, an interaction between WHtR and sex was detected, that is, the association between WHtR and risk of hypertension was stronger in male than in female (male, HR: 1.37, 95% CI: 1.20–1.58; female, HR: 1.28, 95% CI: 1.14–1.43; P for interaction = 0.001).

**Table 4:** Subgroups analyses for the association between WHtR and the incidence of hypertension.

	HR (95% CI)	*p* value	P for interaction
Age			0.102
< 60 years	1.36 (1.23, 1.52)	< 0.001	
≥ 60 years	1.19 (1.03, 1.37)	0.016	
Sex			0.001
Male	1.37 (1.20, 1.58)	< 0.001	
Female	1.28 (1.14, 1.43)	< 0.001	
Suburban or rural village			0.772
Yes	1.29 (1.17, 1.43)	< 0.001	
No	1.24 (0.96, 1.60)	0.100	
Married			0.573
Yes	1.34 (1.21, 1.47)	< 0.001	
No	1.15 (0.82, 1.62)	0.416	
Educational level			0.876
Lower than high school	1.28 (1.16, 1.41)	< 0.001	
High school	1.37 (1.01, 1.85)	0.041	
Higher than high school	1.53 (0.95, 2.47)	0.083	
Smoking status			0.806
Present	1.22 (0.94, 1.58)	0.143	
Past	0.76 (0.30, 1.92)	0.564	
Never	1.29 (1.16, 1.43)	< 0.001	
Diabetes			0.451
Yes	1.38 (0.90, 2.11)	0.135	
No	1.32 (1.20, 1.45)	< 0.001	
Obesity			0.300
Yes	1.13 (0.77, 1.65)	0.537	
No	1.29 (1.16, 1.43)	< 0.001	

The multivariate adjusted model used in the subgroups analysis consisted of all covariates used in the Model 3 in Table 3 except for the variable (as a categorical variable) that was used for stratification. The HR was examined by per 0.1-unit increase of WHtR. The interaction of WHtR and variables used for stratification was examined by likelihood ratio tests. WHtR, waist-to-height ratio; HR, hazard ratio; CI, confidence interval.

**Figure 4:**
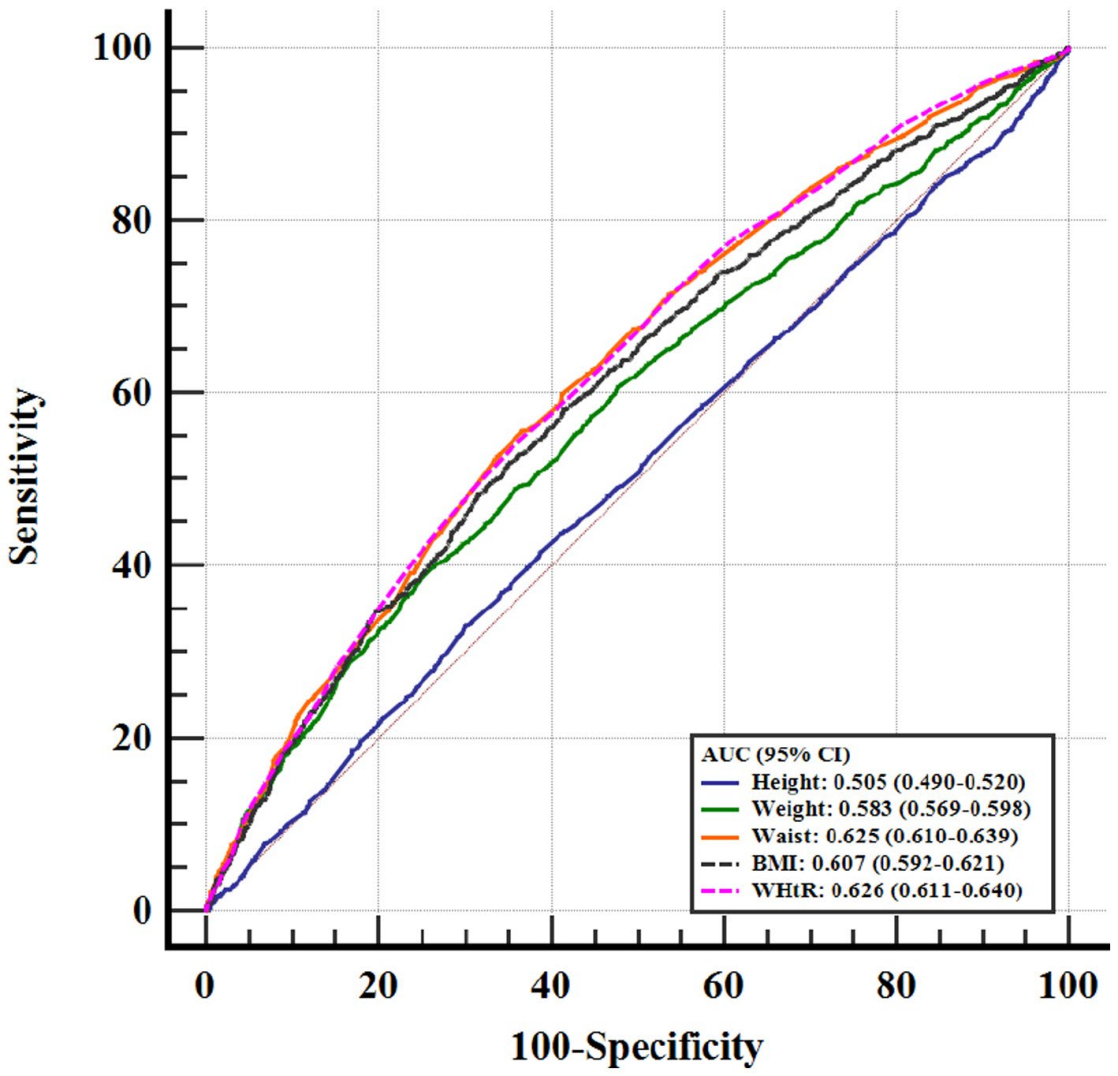
ROC curve evaluating diagnostic performance of different anthropometric indicators for the incidence of hypertension. WHtR, waist-to-height ratio; BMI, body mass index; ROC, receiver operator characteristic; AUC, area under the curve; CI, confidence interval.

**Figure 5:**
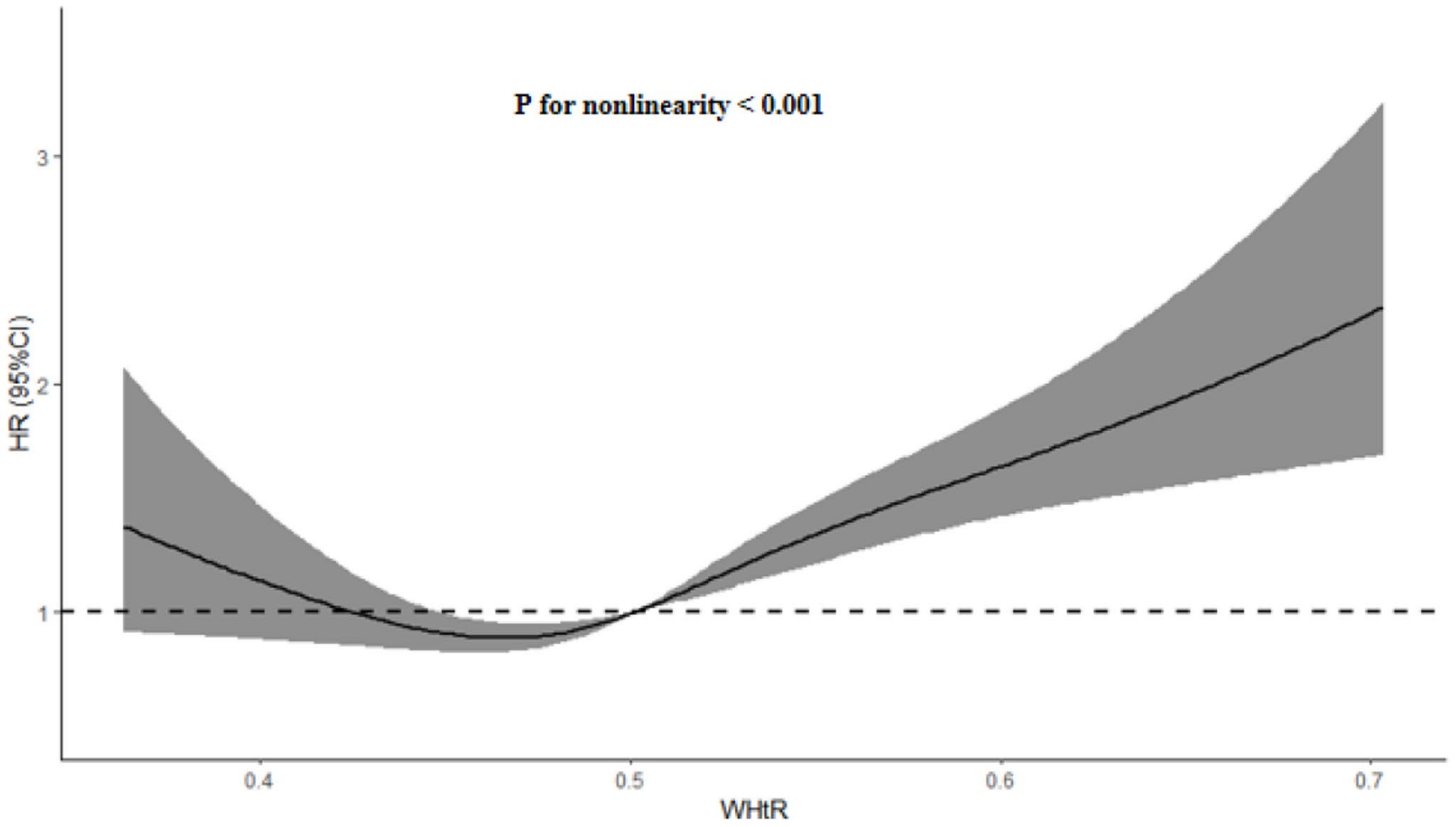
HR (95% CI) for the incidence of hypertension by WHtR. The association was adjusted for variables included in the Model 3 in Table 3. WHtR, waist-to-height ratio; HR, hazard ratio; CI, confidence interval.

Table 5 and Figure 4 presented the C-index of WHtR and other indicators to predict the risk of hypertension. Although WHtR [area under the curve (AUC): 0.626], weight (AUC: 0.583), WC (AUC: 0.625), and BMI (AUC: 0.607) all predicted the occurrence of hypertension, WHtR was superior to weight and BMI in terms of diagnostic performance (P for comparison <0.05). Additionally, as shown in Figure 5, a nonlinear correlation between WHtR and the risk of hypertension was also uncovered (P for nonlinearity <0.001).

**Table 5:** C-index of different anthropometric indicators for predicting hypertension incidence.

Variables	AUC	95% CI	*p* value	P for comparison
WHtR	0.626	0.611–0.640	< 0.001	Reference
Height	0.505	0.490–0.520	0.484	< 0.001
Weight	0.583	0.569–0.598	< 0.001	< 0.001
WC	0.625	0.610–0.639	< 0.001	0.799
BMI	0.607	0.592–0.621	< 0.001	0.009

WHtR, waist-to-height ratio; WC, waist circumference; BMI, body mass index; AUC, area under the curve; CI, confidence interval.

## 4 Discussion

In this community-based survey of the general population, we found that higher levels of WHtR were associated with a higher risk of new-onset hypertension, independent of BMI and other cardiovascular risk factors. Furthermore, we revealed that the correlation between WHtR and the risk of new-onset hypertension was statistically significant in most subgroups, and that WHtR was also superior to BMI in terms of the diagnostic performance to discriminate the occurrence of new-onset hypertension. More importantly, we also detected a nonlinear association between WHtR and the risk of new-onset hypertension, suggesting that when formulating hypertension prevention strategies, we should not only take into account the important impact of WHtR, but also pay attention to the heterogeneity across populations and risk levels.

The burden of CVD caused by obesity is very high, yet the main anthropometric measure of obesity in clinical practice remains BMI, which has a number of limitations. As BMI is derived from a combined height and weight formula, it does not take into account the importance of body fat distribution and may also be affected by different sexes, ages or ethnicities (29–31). Therefore, BMI may be the best indicator of general adiposity, while it may not be a better indicator of central adiposity. In addition, there has also been much interest in the “obesity paradox” involving BMI, which, contrary to initial conclusions about obesity, suggests that obesity does not necessarily shorten a patient’s life expectancy, and in some cases may even be “beneficial.” (24, 32, 33). As a result, a number of alternative anthropometric measures reflecting obesity status have been developed and attempted for CVD risk screening (34, 35).

Currently, the nutritional metrics that have received the most attention besides BMI include WC, WHR and WHtR, with WC being a key indicator of central obesity. In addition, WC and WHR have been widely recognized in the prevention and management of CVD, but the correlation between WHtR and CVD does not seem to be fully recognised and WHtR is not considered superior to BMI and WHR in predicting some diseases in some studies, and the stratified and non-linear associations of WHtR with hypertension have rarely been explored. Therefore, the relevance of WHtR to CVD or cardiometabolic-related disease needs to be more fully explored and discussed, especially for hypertension (36–38). Additionally, WHtR not only takes into account the distribution of body fat but also reduces the effects of sex and ethnic heterogeneity, and has been recommended as an alternative to WC for assessing the severity of obesity (39). Therefore, based on the above background, more and more studies have begun to explore the association between WHtR and hypertension. For example, in a large cohort study based on 13,044 participants, Petermann-Rocha et al. found that WHtR was superior to other anthropometric indicators, including WC, in predicting hypertension risk (14). In addition, Moosaie et al. demonstrated that WHtR was a stronger predictor of hypertension than WHR and BMI in a large longitudinal study involving 1,685 normotensive patients with type 2 diabetes (17). Furthermore, Chen et al. further demonstrated a stronger correlation between higher levels of WHtR and higher risk of hypertension incidence compared to WC and BMI in a study including 9,905 participants from the rural Chinese cohort study (19). These studies suggest that WHtR is not only strongly associated with hypertension risk, but may be superior to WC in its ability to predict hypertension risk (14, 17, 19), while other studies have not validated the superiority of WHtR (16, 18, 40). For example, Rezende et al. did not find WHtR to be superior to WC in predicting hypertension incidence in a small-sample prospective cohort study, and even the AUC of WHtR was lower than that of WC in men and in the total sample (16). Also, Li et al. found a stronger correlation between WC and hypertension incidence than WHtR (18). Additionally, a systematic review and meta-analysis conducted by Lo et al. also confirmed that WHtR was weaker than WC in screening for hypertension (40). Thus, whether WHtR is necessarily superior to WC or other anthropometric measures in predicting hypertension needs to be more fully investigated.

In addition, most studies have only revealed the correlation between WHtR and risk of hypertension without further exploring the question of whether this correlation is a nonlinear one. Fortunately, our study not only reconfirmed the correlation between WHtR and hypertension risk in a Chinese community-based population, but also derived some stratified associations between them, and furthermore, found that the correlation between WHtR and hypertension risk was a non-linear association with an approximate U-shape, that is, both lower levels of WHtR and higher levels of WHtR were strongly associated with higher risk of new-onset hypertension, which was in line with the the finding that both malnutrition and overnutrition are both a risk for human health (41, 42). Furthermore, since the blood pressure levels of some patients with hypertension were controlled at normal levels due to the effectiveness of antihypertensive therapy, the SBP/DBP of these patients may be lower than 140/90 mmHg, which is generally in line with the results observed in our study. However, our study was only a preliminary exploration of the phenotype, and more studies in different populations and races are needed to further validate the stability of the results.

Nonetheless, there were still some limitations in this study. First, because of the lack of genetic data associated with WHtR and hypertension in this study, we were unable to determine a causal association between WHtR and hypertension. Second, in this study, hypertension was assessed based on a combination of participants’ blood pressure measurements, medical history, and medication, rather than being diagnosed during a hospital outpatient or inpatient stay, so there may have been diagnostic bias. In addition, although the height of the participants can remain almost the same during the follow-up, the WC can change significantly with the nutritional status of the participants, and hence the dynamic assessment of WHtR is more scientific and accurate for predicting the risk of hypertension than a single assessment. Finally, despite evidence of a correlation between central obesity and hypertension, the specific pathological mechanisms underlying the correlation between WHtR and hypertension remain unclear (43). We can only speculate that the deleterious effects of high levels of WHtR on hypertension may be attributable to central obesity or overnutrition, whereas the potentially deleterious effects of low levels of WHtR on hypertension may be attributable to debilitation or malnutrition. More observational studies and genetic association studies (such as Mendelian randomised studies) are also needed to further compensate for these limitations in the future.

## 5 Conclusion

In this study, we obtained the following main findings: (1) higher levels of WHtR were associated with a higher risk of hypertension incidence; (2) the correlation between them was stable in most subgroups; and (3) the correlation between them was an approximately U-shaped nonlinearity. These findings suggest that we should pay more attention to the additional effects of anthropometric indicators other than BMI in the management of risk factors for hypertension, and also suggest that we should develop different preventive strategies for different WHtR levels in the daily management of hypertension risk. And our study also suggests that incorporating WHtR into routine screening in clinical practice and epidemiological surveys would be of great interest.

## Data availability statement

Publicly available datasets were analyzed in this study. This data can be found at: https://www.cpc.unc.edu/projects/china.

## Ethics statement

The CHNS was approved by the institutional review committees at the University of North Carolina at Chapel Hill and the National Institute of Nutrition and Food Safety, Chinese Center for Disease Control and Prevention. The study protocol was conducted in accordance with the Declaration of Helsinki. The participants provided their written informed consent to participate in this study.

## Author contributions

ZW: Conceptualization, Data curation, Methodology, Software, Writing – original draft, Writing – review & editing. QS: Data curation, Writing – original draft, Writing – review & editing. XY: Conceptualization, Funding acquisition, Methodology, Project administration, Software, Supervision, Writing – review & editing. JT: Conceptualization, Funding acquisition, Project administration, Supervision, Writing – review & editing. JZ: Conceptualization, Funding acquisition, Project administration, Supervision, Writing – review & editing.

## References

[1] Lewington S Lacey B Clarke R Guo Y Kong XL Yang L. The burden of hypertension and associated risk for cardiovascular mortality in China. JAMA Intern Med. (2016) 176:524–32. doi: 10.1001/jamainternmed.2016.0190, PMID: 26975032

[2] Kearney PM Whelton M Reynolds K Muntner P Whelton PK He J. Global burden of hypertension: analysis of worldwide data. Lancet. (2005) 365:217–23. doi: 10.1016/S0140-6736(05)17741-1 15652604

[3] Miazgowski T Kopec J Widecka K Miazgowski B Kaczmarkiewicz A. Epidemiology of hypertensive heart disease in Poland: findings from the global burden of disease study 2016. Arch Med Sci. (2019) 17:874–80. doi: 10.5114/aoms.2019.85222, PMID: 34336015 PMC8314396

[4] Liu J Bu X Wei L Wang X Lai L Dong C. Global burden of cardiovascular diseases attributable to hypertension in young adults from 1990 to 2019. J Hypertens. (2021) 39:2488–96. doi: 10.1097/HJH.0000000000002958, PMID: 34269332

[5] Wang Z Luo Y Yang S Zuo M Pei R He J. Death burden of high systolic blood pressure in Sichuan Southwest China 1990-2030. BMC Public Health. (2020) 20:406. doi: 10.1186/s12889-020-8377-6, PMID: 32223743 PMC7104502

[6] Qian J Chen Y Lu D Ma J Liu K. The prevalence, disability-adjusted life years, and mortality of hypertensive heart disease and its attributable risk factors: results from the global burden disease study 2019. Arch Med Sci. (2023) 19:1186–200. doi: 10.5114/aoms/169477, PMID: 37732060 PMC10507767

[7] Mansouri A Khosravi A Mehrabani-Zeinabad K Kopec JA Adawi KII Lui M. Trends in the burden and determinants of hypertensive heart disease in the eastern Mediterranean region, 1990-2019: an analysis of the global burden of disease study 2019. EClinicalMedicine. (2023) 60:102034. doi: 10.1016/j.eclinm.2023.102034, PMID: 37396799 PMC10314131

[8] Ren Y Wang Z Wang Q. The trend of hypertension-related chronic kidney disease from 1990 to 2019 and its predictions over 25 years: an analysis of the global burden of disease study 2019. Int Urol Nephrol. (2023). doi: 10.1007/s11255-023-03707-w, PMID: 37542001

[9] Seravalle G Grassi G. Obesity and hypertension. Pharmacol Res. (2017) 122:1–7. doi: 10.1016/j.phrs.2017.05.013 28532816

[10] Beilin LJ Puddey IB Burke V. Lifestyle and hypertension. Am J Hypertens. (1999) 12:934–45. doi: 10.1016/s0895-7061(99)00057-6 10509554

[11] Adhikari B Delgado-Ron JA Van den Bosch M Dummer T Hong A Sandhu J. Community design and hypertension: walkability and park access relationships with cardiovascular health. Int J Hyg Environ Health. (2021) 237:113820. doi: 10.1016/j.ijheh.2021.113820, PMID: 34365293

[12] Lawal Y Mshelia-Reng R Omonua SO Odumodu K Shuaibu R Itanyi UD. Comparison of waist-height ratio and other obesity indices in the prediction of diabetic peripheral neuropathy. Front Nutr. (2022) 9:949315. doi: 10.3389/fnut.2022.949315, PMID: 36276814 PMC9582519

[13] Pasdar Y Moradi S Moludi J Saiedi S Moradinazar M Hamzeh B. Waist-to-height ratio is a better discriminator of cardiovascular disease than other anthropometric indicators in Kurdish adults. Sci Rep. (2020) 10:16228. doi: 10.1038/s41598-020-73224-8, PMID: 33004896 PMC7530727

[14] Petermann-Rocha F Ulloa N Martínez-Sanguinetti MA Leiva AM Martorell M Villagrán M. Is waist-to-height ratio a better predictor of hypertension and type 2 diabetes than body mass index and waist circumference in the Chilean population? Nutrition. (2020) 79-80:110932. doi: 10.1016/j.nut.2020.110932, PMID: 32847773

[15] Park SH Choi SJ Lee KS Park HY. Waist circumference and waist-to-height ratio as predictors of cardiovascular disease risk in Korean adults. Circ J. (2009) 73:1643–50. doi: 10.1253/circj.cj-09-0161, PMID: 19638708

[16] Rezende AC Souza LG Jardim TV Perillo NB Araújo YCL de Souza SG. Is waist-to-height ratio the best predictive indicator of hypertension incidence? A cohort study. BMC Public Health. (2018) 18:281. doi: 10.1186/s12889-018-5177-3, PMID: 29478413 PMC6389116

[17] Moosaie F Fatemi Abhari SM Deravi N Karimi Behnagh A Esteghamati S Dehghani Firouzabadi F. Waist-to-height ratio is a more accurate tool for predicting hypertension than waist-to-hip circumference and BMI in patients with type 2 diabetes: a prospective study. Front Public Health. (2021) 9:726288. doi: 10.3389/fpubh.2021.726288, PMID: 34692623 PMC8529190

[18] Li N Yang T Yu WQ Liu H. Is waist-to-height ratio superior to body mass index and waist circumference in predicting the incidence of hypertension? Ann Nutr Metab. (2019) 74:215–23. doi: 10.1159/000499073, PMID: 30889583

[19] Chen X Liu Y Sun X Yin Z Li H Deng K. Comparison of body mass index, waist circumference, conicity index, and waist-to-height ratio for predicting incidence of hypertension: the rural Chinese cohort study. J Hum Hypertens. (2018) 32:228–35. doi: 10.1038/s41371-018-0033-6, PMID: 29416119

[20] Yan S Li J Li S Zhang B Du S Gordon-Larsen P. The expanding burden of cardiometabolic risk in China: the China health and nutrition survey. Obes Rev. (2012) 13:810–21. doi: 10.1111/j.1467-789X.2012.01016.x, PMID: 22738663 PMC3429648

[21] Popkin BM Du S Zhai F Zhang B. Cohort profile: the China health and nutrition survey--monitoring and understanding socio-economic and health change in China, 1989-2011. Int J Epidemiol. (2010) 39:1435–40. doi: 10.1093/ije/dyp322, PMID: 19887509 PMC2992625

[22] American Diabetes Association. 2 classification and diagnosis of diabetes. Diabetes Care. (2015) 38:S8–S16. doi: 10.2337/dc15-S005 25537714

[23] Cox BD Whichelow MJ Ashwell M Prevost AT Lejeune SR. Association of anthropometric indices with elevated blood pressure in British adults. Int J Obes Relat Metab Disord. (1997) 21:674–80. doi: 10.1038/sj.ijo.0800459, PMID: 15481767

[24] Elagizi A Kachur S Lavie CJ Carbone S Pandey A Ortega FB. An overview and update on obesity and the obesity paradox in cardiovascular diseases. Prog Cardiovasc Dis. (2018) 61:142–50. doi: 10.1016/j.pcad.2018.07.003, PMID: 29981771

[25] Wang L Zhou B Zhao Z Yang L Zhang M Jiang Y. Body-mass index and obesity in urban and rural China: findings from consecutive nationally representative surveys during 2004-18. Lancet. (2021) 398:53–63. doi: 10.1016/S0140-6736(21)00798-4 34217401 PMC7617101

[26] Rabi DM McBrien KA Sapir-Pichhadze R Nakhla M Ahmed SB Dumanski SM. Hypertension Canada's 2020 comprehensive guidelines for the prevention, diagnosis, risk assessment, and treatment of hypertension in adults and children. Can J Cardiol. (2020) 36:596–624. doi: 10.1016/j.cjca.2020.02.086, PMID: 32389335

[27] Guo J Zhu YC Chen YP Hu Y Tang XW Zhang B. The dynamics of hypertension prevalence, awareness, treatment, control and associated factors in Chinese adults: results from CHNS 1991-2011. J Hypertens. (2015) 33:1688–96. doi: 10.1097/HJH.0000000000000594, PMID: 26136071

[28] Zhang X Ye R Sun L Liu X Wang S Meng Q. Relationship between novel anthropometric indices and the incidence of hypertension in Chinese individuals: a prospective cohort study based on the CHNS from 1993 to 2015. BMC Public Health. (2023) 23:436. doi: 10.1186/s12889-023-15208-7, PMID: 36879238 PMC9990350

[29] Heymsfield SB Peterson CM Thomas DM Heo M Schuna JM Jr. Why are there race/ethnic differences in adult body mass index-adiposity relationships? A quantitative critical review. Obes Rev. (2016) 17:262–75. doi: 10.1111/obr.12358, PMID: 26663309 PMC4968570

[30] Okorodudu DO Jumean MF Montori VM Romero-Corral A Somers VK Erwin PJ. Diagnostic performance of body mass index to identify obesity as defined by body adiposity: a systematic review and meta-analysis. Int J Obes. (2010) 34:791–9. doi: 10.1038/ijo.2010.5, PMID: 20125098

[31] Rothman KJ. BMI-related errors in the measurement of obesity. Int J Obes. (2008) 32:S56–9. doi: 10.1038/ijo.2008.87, PMID: 18695655

[32] Lavie CJ Milani RV Ventura HO. Obesity and cardiovascular disease: risk factor, paradox, and impact of weight loss. J Am Coll Cardiol. (2009) 53:1925–32. doi: 10.1016/j.jacc.2008.12.068 19460605

[33] Artham SM Lavie CJ Milani RV Ventura HO. The obesity paradox: impact of obesity on the prevalence and prognosis of cardiovascular diseases. Postgrad Med. (2008) 120:34–41. doi: 10.3810/pgm.2008.07.1788 18654066

[34] Loehr LR Rosamond WD Poole C McNeill AM Chang PP Folsom AR. Association of multiple anthropometrics of overweight and obesity with incident heart failure: the atherosclerosis risk in communities study. Circ Heart Fail. (2009) 2:18–24. doi: 10.1161/CIRCHEARTFAILURE.108.813782, PMID: 19808311 PMC2748859

[35] Butt JH Petrie MC Jhund PS Sattar N Desai AS Køber L. Anthropometric measures and adverse outcomes in heart failure with reduced ejection fraction: revisiting the obesity paradox. Eur Heart J. (2023) 44:1136–53. doi: 10.1093/eurheartj/ehad083, PMID: 36944496 PMC10111968

[36] Haufs MG Zöllner YF. Waist-hip ratio more appropriate than body mass index. Dtsch Arztebl Int. (2020) 117:659. doi: 10.3238/arztebl.2020.0659a, PMID: 33357347 PMC7829451

[37] Huxley R Mendis S Zheleznyakov E Reddy S Chan J. Body mass index, waist circumference and waist:hip ratio as predictors of cardiovascular risk--a review of the literature. Eur J Clin Nutr. (2010) 64:16–22. doi: 10.1038/ejcn.2009.68 19654593

[38] de Koning L Merchant AT Pogue J Anand SS. Waist circumference and waist-to-hip ratio as predictors of cardiovascular events: meta-regression analysis of prospective studies. Eur Heart J. (2007) 28:850–6. doi: 10.1093/eurheartj/ehm026, PMID: 17403720

[39] Ashwell M Gibson S. Waist-to-height ratio as an indicator of 'early health risk': simpler and more predictive than using a 'matrix' based on BMI and waist circumference. BMJ Open. (2016) 6:e010159. doi: 10.1136/bmjopen-2015-010159, PMID: 26975935 PMC4800150

[40] Lo K Wong M Khalechelvam P Tam W. Waist-to-height ratio, body mass index and waist circumference for screening paediatric cardio-metabolic risk factors: a meta-analysis. Obes Rev. (2016) 17:1258–75. doi: 10.1111/obr.12456, PMID: 27452904

[41] Bellanti F Lo Buglio A Quiete S Vendemiale G. Malnutrition in hospitalized old patients: screening and diagnosis, clinical outcomes, and management. Nutrients. (2022) 14:910. doi: 10.3390/nu14040910, PMID: 35215559 PMC8880030

[42] Kim M Basharat A Santosh R Mehdi SF Razvi Z Yoo SK. Reuniting overnutrition and undernutrition, macronutrients, and micronutrients. Diabetes Metab Res Rev. (2019) 35:e3072. doi: 10.1002/dmrr.3072, PMID: 30171821

[43] Das S Goswami V Chandel S. Normal weight central obesity and hypertension in India: cross-sectional finding from LASI, 2017-19. Nutr Metab Cardiovasc Dis. (2023) 33:1888–98. doi: 10.1016/j.numecd.2023.06.022, PMID: 37544873

